# Systemic immune-inflammation index as a predictor of prognosis after carotid artery stenting compared with C-reactive protein

**DOI:** 10.1371/journal.pone.0288564

**Published:** 2023-07-13

**Authors:** Shuji Morikawa, Kenji Okumura, Naoya Inoue, Takashi Ogane, Yohei Takayama, Toyoaki Murohara

**Affiliations:** 1 Department of Cardiology, Chutoen General Medical Center, Kakegawa, Shizuoka, Japan; 2 Department of Cardiology, Nagoya University Graduate School of Medicine, Showa-ku, Nagoya, Japan; 3 Department of Cardiology, Tohno Kosei Hospital, Mizunami, Japan; Osaka University Graduate School of Medicine, JAPAN

## Abstract

**Background:**

Immune-inflammatory processes are highly associated with the progression of atherosclerosis. The systemic immune-inflammation index (SII) is a potential predictor for clinical outcomes in patients with stroke and ischemic heart disease. Therefore, this study aimed to investigate whether SII can accurately predict the short- and long-term prognoses in patients who underwent carotid artery stenting (CAS) compared to that with C-reactive protein (CRP).

**Methods:**

This study was a single-center retrospective investigation. Overall, 129 patients who underwent CAS were categorized into tertiles based on their SII levels. We primarily investigated the long-term major adverse cardiac and cerebrovascular events (MACCE) and secondarily the in-hospital and long-term stroke incidence, as well as all-cause death.

**Results:**

The in-hospital stroke rate tended to increase with a rise in SII (*P* = 0.13). Over the 5-year follow-up period, the Kaplan–Meier overall incidence of MACCE was 9.3%, 16.3%, and 39.5% in the lowest to highest tertiles, respectively (log-rank trend test, *P*<0.001). The rates of stroke and MACCE during the long-term follow-up were significantly higher with increasing SII. Cox regression analysis showed that the highest tertile of SII (>647) was a predictor of the incidence of long-term stroke (hazard ratio (HR), 21.3; 95% confidence interval (CI), 2.41–188; *P* = 0.006) and MACCE (HR, 3.98; 95% CI, 1.80–8.81; *P*<0.001). However, after adjusting for both SII and CRP, only SII remained a significant independent predictor, whereas CRP became less relevant. The receiver operating characteristic curve analysis of long-term MACCE showed that the area under the curve (AUC) for SII (AUC, 0.72; 95% CI, 0.60–0.84; *P*<0.001) was greater than that of CRP (AUC, 0.64; 95% CI, 0.51–0.77; *P* = 0.040).

**Conclusion:**

SII was shown to be an independent predictor of long-term prognosis in patients who underwent CAS and was suggested to be superior to CRP as an inflammatory prognosis predictor.

## Introduction

Stroke causes significant morbidity and mortality worldwide, with atherosclerosis of the carotid artery causing approximately 10–20% of all ischemic strokes [1]. Therefore, to prevent stroke, high-risk patients have been undergoing carotid artery stenting (CAS) [2]. However, the long-term mortality and major adverse cardiac and cerebrovascular events (MACCE) of patients undergoing CAS are affected by age, diabetes mellitus (DM), and neurological symptoms before CAS [3, 4]. Moreover, factors including age, symptomatic status, chronic kidney disease, and DM are associated with stroke and death within 1 month after CAS [5]. Therefore, among the availability of various predictors, it is crucial to identify an easily identifiable predictor for short- and long-term prognoses in patients who have undergone CAS.

Atherosclerosis is widely recognized as an inflammatory disease characterized by intravascular lipids and associated with immune responses during its progression [6]. High-sensitive C-reactive protein (CRP) is the most widely used inflammatory marker and is recognized as a useful predictor of cardiovascular disease [7] and stroke [8]. Furthermore, several inflammatory markers have been proven as useful predictors of myocardial infarction [9]. Some studies have explored the use of novel hematological markers as potential predictors, such as the pre-procedural neutrophil/albumin, which is a predictor of in-stent restenosis of CAS [10].

The systemic immune-inflammation index (SII), which consists of lymphocyte, neutrophil, and platelet counts, is a novel index that is a predictor of poor outcomes in patients with cancer [11], cardiovascular disease [12], and acute ischemic stroke [13]. Although only one study has reported that SII may influence clinical outcomes in patients undergoing CAS [14], it is not yet clear whether SII is a stronger predictor than traditional inflammatory markers such as CRP in this context. Therefore, this study aimed to compare the ability of SII to predict the short-and long-term prognoses in patients who underwent CAS with that of CRP.

## Materials and methods

### Patients

Data from the Chutoen General Medical Center between January 27, 2011, and July 27, 2017, were analyzed in this single-center retrospective study. Overall, 141 consecutive patients who underwent CAS at this hospital during this period were enrolled. The inclusion criteria were ≥50% carotid artery stenosis in symptomatic patients or ≥80% stenosis in asymptomatic patients [15]. A symptomatic lesion was defined as the presence of a transient ischemic attack or ischemic stroke within the preceding 6 months [16]. The exclusion criteria were as follows: (1) patients aged >85 years; (2) those with systemic inflammatory disease; (3) those with malignancy; (4) those with liver failure; and/or (5) those with end-stage renal failure. Overall, 129 patients met the eligibility criteria for the study, whereas 12 were excluded. The study was approved by the Ethics Committee of Chutoen General Medical Center (approval number: KENI 217) and adhered to the principles of the Declaration of Helsinki. Informed consent was not required because of the retrospective nature of the study.

### Data collection

Data for this study population were recorded, including arteriosclerosis risk factors, clinical examination results, and procedure data. Hypertension was diagnosed according to the administration of hypotensive drugs or systolic blood pressure of ≥140 mmHg and/or diastolic blood pressure of ≥90 mmHg. DM was diagnosed according to the administration of antidiabetic drugs or both glycated hemoglobin of ≥6.5% and fasting glucose levels of ≥126 mg/dL or non-fasting glucose levels of ≥200 mg/dL. Hyperlipidemia was defined as a low-density lipoprotein cholesterol level (LDL-C) of ≥140 mg/dL or administering hypolipidemic medications. Blood sampling was performed before the CAS procedure, and hematological findings were measured using standard laboratory techniques. SII was defined as peripheral platelet count × neutrophil/lymphocyte count [11].

### CAS procedure

CAS was performed in all patients according to the current guidelines [17]. The operator appropriately selected the self-expandable stent, balloon, and embolism protection device. Aspirin 100 mg and clopidogrel 75 mg were administered for at least 1 week before and 1–2 months after CAS, after which single antiplatelet therapy was continued.

### Endpoints and follow-up

This study’s primary endpoint was the incidence of long-term (from discharge to 5-year follow-up) MACCE, which included all-cause mortality, stroke, and myocardial infarction. The secondary endpoints were the incidence of stroke both in-hospital and during the long-term follow-up, and all-cause death. Data were collected from hospital records and through phone calls to patients or their families.

### Statistical analysis

Normally distributed continuous variables were reported as mean ± standard deviation and analyzed using one-way variance analysis. Non-normally distributed variables were reported as medians (quartiles) and assessed using the Kruskal–Wallis test. Categorical variables were reported as numbers (percentages) and analyzed using the chi-square or Fisher’s exact tests when appropriate. The incidence of MACCE among the three groups was calculated after a 5-year follow-up using the Kaplan–Meier method and analyzed using the log-rank trend test. The Cox regression analysis was used to assess whether SII and CRP were independent predictors, adjusting for the previously identified confounders [4, 18]. Statistical significance was set at P < 0.05.

In addition, the correlation between SII and CRP was assessed using Spearman’s rank correlation coefficient. The area under the curve (AUC) of the receiver operating characteristic (ROC) curves was used to compare the abilities of SII and CRP to detect long-term MACCE. All statistical analyses were performed using SPSS (IBM SPSS Statistics; Version 28) and EZR (Saitama Medical Center, Jichi Medical University, Saitama, Japan).

## Results

A total of 129 patients who had undergone CAS were enrolled. All patients had a complete 5-year follow-up upon discharge and were categorized into tertile (T) groups based on their SII levels that increased from tertile 1 (T1) to tertile 3 (T3). Table 1 presents the patients’ baseline characteristics. No significant differences were found among the groups for age, sex, symptomatic lesion, arteriosclerosis risk factors, history of coronary artery disease, or procedure. The blood test results were similar among the groups, including hemoglobin levels, creatinine levels, estimated glomerular filtration rate (eGFR), and lipid tests. However, the T3 group had the highest leukocyte, neutrophil, and platelet counts and the highest CRP levels among the three groups, but the lowest lymphocyte count. Furthermore, all groups had similar usage of statins.

**Table 1:** Baseline characteristics of the study patients according to SII tertiles.

	Tertile 1	Tertile 2	Tertile 3	*P-*value
	SII<435	435≤SII≤647	SII>647	
	(n = 43)	(n = 43)	(n = 43)	
**Age (years)**	73±6	71±7	72±6	0.24
**Sex, male, n (%)**	40 (93.0)	39 (90.7)	40 (93.0)	0.90
**Symptomatic lesion, n (%)**	14 (32.6)	15 (34.9)	20 (46.5)	0.36
**Risk factors, n (%)**				
** Hypertension**	31 (72.1)	30 (69.8)	34 (79.1)	0.60
** Diabetes mellitus**	18 (41.9)	13 (30.2)	18 (41.9)	0.44
** Hyperlipidemia**	20 (46.5)	24 (55.8)	25 (58.1)	0.52
** Previous or current smokers**	27 (62.8)	26 (60.5)	29 (67.4)	0.79
**Previous myocardial infarction**	8 (18.6)	5 (11.6)	12 (27.9)	0.18*
**Previous PCI**	15 (34.9)	11 (25.6)	18 (41.9)	0.28
**Previous CABG**	0 (0)	1 (2.3)	3 (7.0)	0.32*
**Procedure, n (%)**				
** Proximal protection**	20 (46.5)	21 (48.8)	18 (41.9)	0.80
** Distal protection**	23 (53.5)	22 (51.2)	25 (58.1)	0.80
** Predilatation**	30 (69.8)	32 (74.4)	25 (51.8)	0.25
** Postdilatation**	42 (97.7)	42 (97.7)	43 (100)	0.60
**Laboratory findings**				
** White blood cell count, /μl**	5760±1523	5837±1280	6711±1543	0.003
** Neutrophil count, /μl**	3217±929	3715±907	4526±885	<0.001
** Lymphocyte count, /μl**	1846±626	1434±338	1284±364	<0.001
** Platelet count, ×10** ^**4**^ **/μl**	17.6±4.4	20.4±3.6	23.1±5.3	<0.001
** SII**	311±77	525±58	822±146	<0.001
** Hemoglobin, g/dl**	12.8±1.8	13.1±1.5	12.8±1.4	0.62
** C-reactive protein, mg/dl**	0.04 (0.02–0.09)	0.05 (0.02–0.10)	0.12 (0.05–0.24)	<0.001
** Creatinine, mg/dl**	1.00±0.33	0.88±0.20	0.99±0.31	0.11
** eGFR, ml/min/1.73 m** ^**2**^	60.4±18.4	66.7±14.1	62.2±19.6	0.23
** LDL-C, mg/dl**	81±29	79±19	85±22	0.52
** HDL-C, mg/dl**	50±13	51±16	47±13	0.22
** Triglycerides, mg/dl**	100±49	91±30	109±51	0.14
**Use of drugs at admission**				
** Statins, n (%)**	34 (79.1)	35 (81.4)	36 (83.7)	0.86

CAS, carotid artery stenting; PCI, percutaneous coronary intervention; CABG, coronary artery bypass graft; SII, systemic immune-inflammation index; eGFR, estimated glomerular filtration rate; LDL-C, low-density lipoprotein cholesterol; HDL-C, high-density lipoprotein cholesterol.

*Data are analyzed using Fisher’s exact tests.

The incidence rate of in-hospital stroke tended to increase from T1 to T3 group, but no statistical significance was found because of only five events (Table 2).

**Table 2:** In-hospital and long-term outcomes according to SII tertiles.

	Tertile 1	Tertile 2	Tertile 3	*P*-value
	**(n = 43)**	**(n = 43)**	**(n = 43)**	
**In-hospital outcome, n (%)**				
** Stroke**	0	1 (2.3)	4 (9.3)	0.13*
**Long-term outcomes, n (%)**				
** Stroke**	0	1 (2.3)	8 (18.6)	0.002*
** All-cause death**	3 (7.0)	6 (14.0)	9 (20.9)	0.19*
** MACCE**	4 (9.3)	7 (16.3)	17 (39.5)	0.002*

CAS, carotid artery stenting; SII, systemic immune-inflammation index; MACCE, major adverse cardiac and cerebrovascular events.

*Data are analyzed using Fisher’s exact tests.

**Fig 1:**
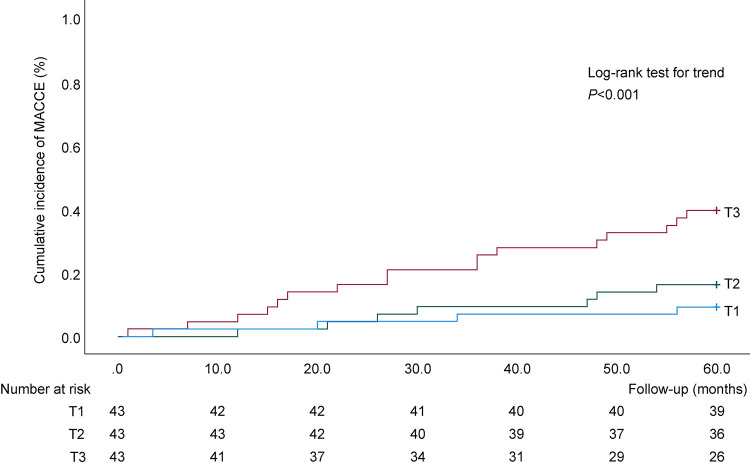
The 5-year Kaplan–Meier overall incidence of MACCE rates according to pre-procedure SII tertiles (T). MACCE, major adverse cardiac and cerebrovascular events; T1, T2, T3 in order from the lowest to highest tertiles of SII; SII, systemic immune-inflammation index.

Regarding the long-term outcomes, the rates of stroke and MACCE increased from the T1 to T3 group, and higher SII levels were associated with a greater incidence (*P* = 0.002 and *P* = 0.002, respectively). No incident cases of stroke were found during the long-term follow-up in the T1 group. However, one and eight incident cases of stroke were observed in the T2 and T3 groups, respectively. Similarly, the incidence of MACCE in the T3 group was approximately 4- and 2.5-fold higher than that in the T1 and T2 groups, respectively. However, only a tendency for an increase in all-cause death was found (*P* = 0.19). As depicted in Fig 1, the Kaplan–Meier curve showed an increase in the cumulative incidence of MACCE according to the high values of SII (log-rank trend test, *P*<0.001).

In the Cox regression analysis adjusted for the previously identified confounders [4, 18], such as age, sex, DM, and eGFR, the highest tertile of SII (>647) was associated with the incidence of stroke (hazard ratio (HR), 21.3; 95% confidence interval (CI), 2.41–188, *P* = 0.006) and MACCE (HR, 3.98; 95% CI, 1.80–8.81, *P*<0.001) during the long-term follow-up period compared with the other patients (Tables 3 and 4). Furthermore, when SII was replaced with CRP, the highest tertile of CRP was also associated with the incidence of stroke (HR, 16.1; 95% CI, 1.97–132, *P* = 0.009) and MACCE (HR, 2.56; 95% CI, 1.19–5.53, *P* = 0.017) during the long-term follow-up. However, after adjusting for both SII and CRP, only SII was found to be an independently significant predictor (Tables 3 and 4), whereas CRP became less relevant.

**Table 3:** Cox regression analysis of long-term stroke incidence.

Long-term stroke incidence			
	Hazard Ratio	95% CI	*P*-value
**Age ≥75 years**	2.61	0.62–11.0	0.19
**Male**	0.39	0.037–4.16	0.44
**Diabetes mellitus**	2.46	0.60–10.0	0.21
**eGFR<60, ml/min/1.73 m** ^**2**^	3.60	0.62–20.8	0.15
**SII (the highest tertile)**	21.3	2.41–188	0.006
**Long-term stroke incidence**			
	**Hazard Ratio**	**95% CI**	***P*-value**
**Age ≥75 years**	1.12	0.28–4.47	0.87
**Male**	0.45	0.054–3.78	0.46
**Diabetes mellitus**	2.00	0.53–7.52	0.31
**eGFR<60, ml/min/1.73 m** ^**2**^	3.13	0.60–16.3	0.18
**CRP (the highest tertile)**	16.1	1.97–132	0.009
**Long-term stroke incidence**			
	**Hazard Ratio**	**95% CI**	***P*-value**
**Age ≥75 years**	1.89	0.46–7.82	0.38
**Male**	0.30	0.027–3.34	0.33
**Diabetes mellitus**	2.31	0.59–9.10	0.23
**eGFR<60, ml/min/1.73 m** ^**2**^	2.96	0.48–18.2	0.24
**SII (the highest tertile)**	10.8	1.19–97.3	0.034
**CRP (the highest tertile)**	7.95	0.92–69.0	0.060

SII, systemic immune-inflammation index; eGFR, estimated glomerular filtration rate; CRP, C-reactive protein; CI, confidence interval.

**Table 4:** Cox regression analysis for long-term incidence of MACCE.

Long-term MACCE			
	Hazard Ratio	95% CI	*P*-value
**Age ≥75 years**	3.15	1.38–7.16	0.006
**Male**	1.22	0.28–5.26	0.79
**Diabetes mellitus**	1.73	0.80–3.74	0.17
**eGFR<60, ml/min/1.73 m** ^**2**^	2.28	0.98–5.29	0.055
**SII (the highest tertile)**	3.98	1.80–8.81	<0.001
**Long-term MACCE**			
	**Hazard Ratio**	**95% CI**	***P*-value**
**Age ≥75 years**	2.14	0.96–4.78	0.063
**Male**	1.24	0.29–5.33	0.77
**Diabetes mellitus**	1.44	0.68–3.03	0.34
**eGFR<60, ml/min/1.73 m** ^**2**^	2.42	1.03–5.66	0.043
**CRP (the highest tertile)**	2.56	1.19–5.53	0.017
**Long-term MACCE**			
	**Hazard Ratio**	**95% CI**	***P*-value**
**Age ≥75 years**	2.83	1.24–6.50	0.014
**Male**	1.09	0.25–4.71	0.91
**Diabetes mellitus**	1.68	0.78–3.62	0.19
**eGFR<60, ml/min/1.73 m** ^**2**^	2.09	0.88–4.92	0.093
**SII (the highest tertile)**	3.31	1.43–7.63	0.005
**CRP (the highest tertile)**	1.78	0.78–4.04	0.17

MACCE, major adverse cardiac and cerebrovascular events; SII, systemic immune-inflammation index; eGFR, estimated glomerular filtration rate; CRP, C-reactive protein; CI, confidence interval.

**Fig 2:**
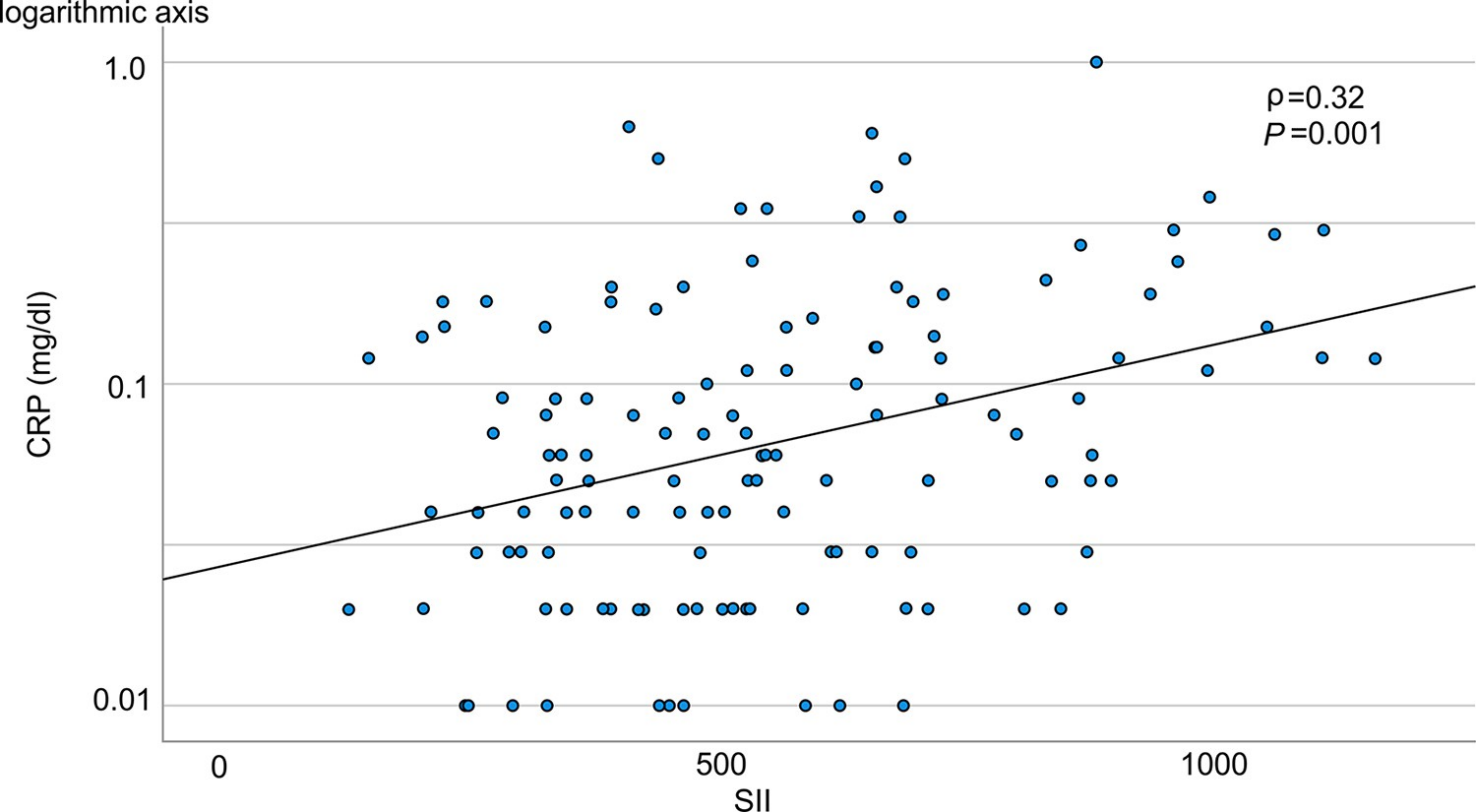
Scatter plot showing a mild positive correlation between SII and CRP. SII, systemic immune-inflammation index; CRP, C-reactive protein.

**Fig 3:**
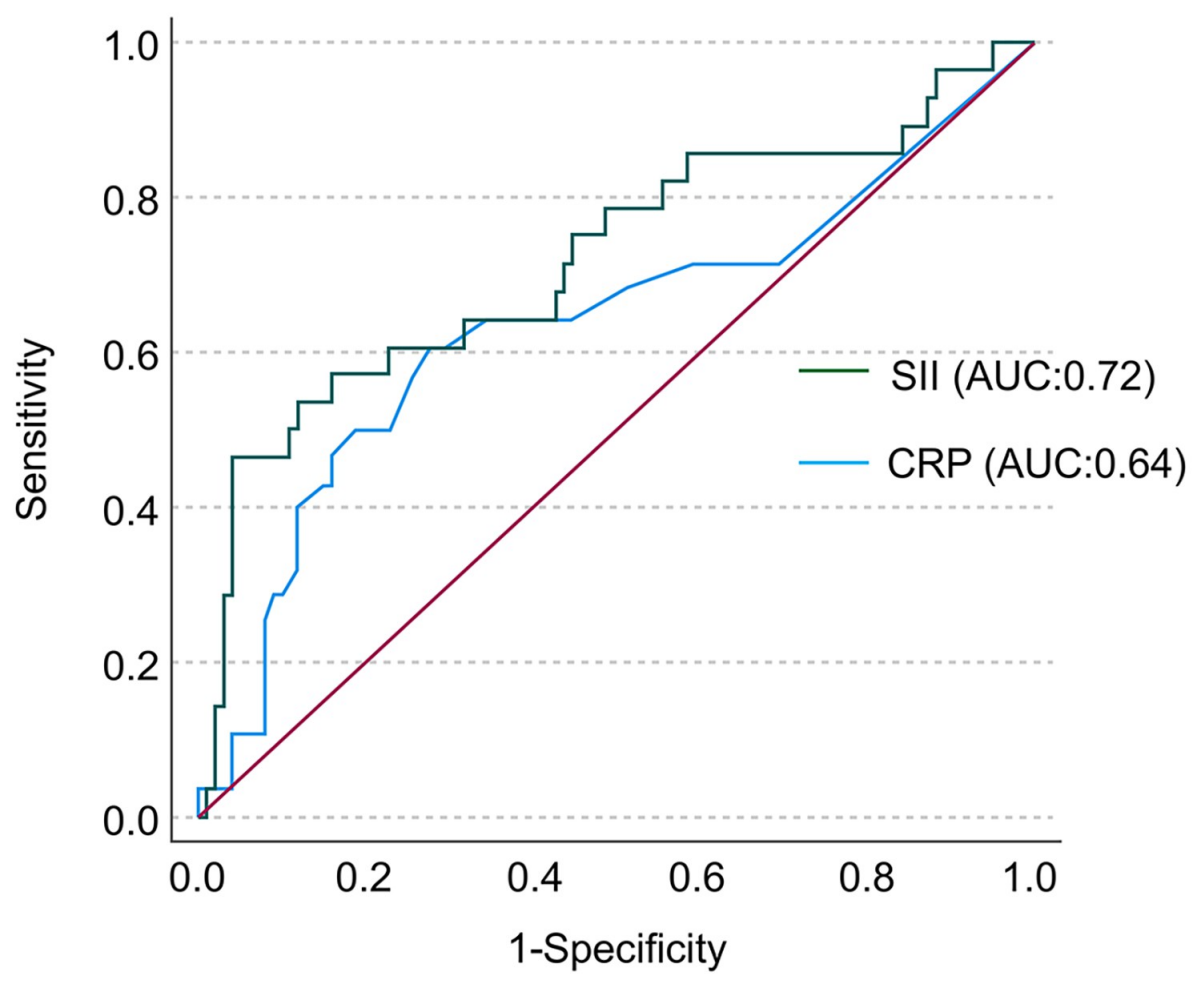
ROC curve analysis of SII and CRP as a predictor of long-term MACCE incidence. ROC, receiver operating characteristics; CRP, C-reactive protein; MACCE, major adverse cardiac and cerebrovascular events.

Fig 2 illustrates the association between SII and CRP. The scatter plot shows a mild, positive correlation (ρ = 0.32, *P* = 0.001); however, some variability exists, and the relationship is not perfectly linear. ROC analysis of long-term MACCE showed that the AUC of SII (AUC, 0.72; 95% CI, 0.60–0.84; *P*<0.001) was greater than that of CRP (AUC, 0.64; 95% CI, 0.51–0.77; *P* = 0.040) (Fig 3), suggesting that SII was superior to CRP as a predictor for long-term MACCE after CAS procedure, but the difference did not reach statistical significance (P = 0.22).

## Discussion

This single-center retrospective cohort study reports that SII, which is a novel surrogate index and comprehensive systematic inflammatory marker, is an independent predictor of stroke and MACCE during the long-term follow-up period in patients who underwent CAS. Moreover, SII was found to be a more useful predictor of long-term MACCE than CRP. To the best of our knowledge, this is the first study to compare the effect of SII and CRP on the long-term prognosis of patients who underwent CAS.

Cardiovascular and cerebrovascular diseases are the major causes of death worldwide, with most cases being attributed to atherosclerosis. Chronic inflammation, which occurs during the pathogenesis of atherosclerosis, pivotally contributes to the progression of these diseases [19]. Atherosclerosis is characterized by lipid accumulation, smooth muscle cell proliferation, and local inflammation, whereas atherosclerotic plaque formation and progression primarily involve inflammation and the immune system [20]. Several studies have shown that inflammatory markers are elevated in atherosclerotic cardiovascular and cerebrovascular diseases [7–9].

Among them, we investigated SII in patients who underwent CAS. SII is a combination of both the neutrophil-to-lymphocyte [21] and the platelet-to-lymphocyte ratio [21], which can be easily and inexpensively determined from routine blood analysis. It is a non-invasive and simple test that is a valid indicator of poor outcomes in certain tumors, such as hepatocellular carcinoma [11]. Subsequent studies have also reported that SII is associated with prognosis in patients with cardiovascular and cerebrovascular diseases [12, 13]. Several studies have investigated the effectiveness of SII to date; however, only one has examined its potential as a prognostic indicator in patients who have undergone CAS [14].

Neutrophils are heavily involved in atherosclerosis development. Furthermore, they are associated with plaque rupture, instability, and reperfusion injury and are involved in the pathophysiological mechanisms of cardiovascular inflammation, proliferation, remodeling, and repair processes [22]. Specifically, acute coronary syndrome (ACS) is caused by the widespread activation of neutrophils due to the presence of vulnerable coronary plaque preceding rupture and erosion before the event [23]. An elevated neutrophil count increases the risk of cardiovascular disease, cerebrovascular disease, and mortality [24, 25]. On the other hand, lymphopenia is observed in inflammatory processes [26] and is linked to higher mortality in patients with acute myocardial infarction [27]. Lymphocyte counts correlated negatively with cardiovascular prognosis. However, platelets play a central role in thrombus formation, and several molecular pathways have been suggested to involve platelets in developing atherogenesis. Both high and low platelet counts are also linked to greater mortality in patients with ACS [28].

We analyzed the patients who underwent CAS according to a previous report, which demonstrated that SII was independently associated with the clinical outcomes of stroke, myocardial infarction, and MACCE in patients who had undergone CAS [14]. Similarly, this study found that SII was an independent prognostic predictor of future stroke incidence and MACCE for 5 years after the CAS procedure; however, no statistical differences were found in mortality rates. SII reflects systemic immune-inflammation status, and chronic inflammation contributes to atherosclerosis, which may explain the association between SII and long-term prognosis in patients who underwent CAS. Although the exact reasons for the poor prognosis among patients undergoing CAS are not yet fully elucidated, chronic inflammation may be involved.

This study shows that SII may be superior to CRP in predicting long-term MACCE in patients who underwent CAS. Although CRP has previously been reported as a predictor of outcomes within 1 month after CAS [29], another previous study did not address the predictive ability of long-term prognosis of other inflammatory markers, such as CRP, compared with SII in patients who underwent CAS [30]. CRP is the most widely used inflammatory marker, and elevated CRP has also been linked to all-cause and cardiovascular mortality in the general population [31]. Although Patrick et al. [30] showed that CRP tended to predict future events in patients who underwent CAS, their study failed to demonstrate a significant association with long-term MACCE, which differs from the definition used in this study. It is unclear why SII may be a better predictor of long-term MACCE than CRP in patients who underwent CAS. Although both SII and CRP were relatively good predictors in this study, they differ in their ability to predict long-term prognosis in patients who underwent CAS. Combining SII and CRP may improve the predictive accuracy for long-term incidence of stroke and MACCE in patients who underwent CAS, as suggested in Tables 3 and 4.

Stroke is one of the most significant perioperative complications that can occur after undergoing CAS. Inflammation-induced destabilization of arteriosclerosis increases the risk of distal embolization during CAS, potentially leading to stroke. Elevated pre-procedure neutrophil counts have been identified as an independent predictor of more frequent distal embolization and stroke during CAS [32]. These findings indicate that systemic inflammation may affect the incidence of periprocedural stroke in patients who have undergone CAS [14]. However, the impact of SII on predicting the incidence of in-hospital stroke was not significant in this study, which could be attributed to the limited sample size.

This study had some limitations. First, the study design was observational and included a relatively small number of patients and events, which may have limited the ability to fully adjust for potential confounding factors in the multivariate analysis. Second, this was a single-center retrospective study; therefore, a larger, prospective, multicenter study is warranted to further evaluate the efficacy of SII or CRP as a prognostic marker in patients who underwent CAS.

### Conclusions

This study demonstrated that an increased SII was a significant independent predictor of long-term MACCE and stroke incidence in patients who underwent CAS. Furthermore, SII was superior to CRP as an inflammatory indicator of prognosis associated with cardiovascular and cerebrovascular diseases. Summarily, SII is a useful index because important information for prognosis can be obtained from a pre-procedural routine blood test and can help identify high-risk patients about to undergo CAS.

## Supporting information

S1 Table Anonymous dataset of 129 patients. (XLSX)

## References

[1] Flaherty ML, Kissela B, Khoury JC, Alwell K, Moomaw CJ, Woo D, et al. Carotid artery stenosis as a cause of stroke. Neuroepidemiology. 2013;40: 36–41. doi: 10.1159/000341410 23075828 PMC3626492

[2] Munich SA, Cress MC, Krishna C, Levy EI. Indications and therapeutic management of carotid stenosis in high-risk patients: SAPPHIRE and beyond. J Neurosurg Sci. 2015;59: 63–71. 25423134

[3] de Donato G, Setacci C, Deloose K, Peeters P, Cremonesi A, Bosiers M. Long-term results of carotid artery stenting. J Vasc Surg. 2008;48: 1431–1440; discussion 1440–1441. doi: 10.1016/j.jvs.2008.07.012 18848755

[4] Arif S, Wojtasik J, Dziewierz A, Bartuś K, Dudek D, Bartuś S. Long-term mortality and follow-up after carotid artery stenting. Hippokratia. 2016;20: 204–208. 29097886 PMC5654437

[5] Khan M, Qureshi AI. Factors associated with increased rates of post-procedural stroke or death following carotid artery stent placement: a systematic review. J Vasc Interv Neurol. 2014;7: 11–20. 24920983 PMC4051899

[6] Ma SD, Mussbacher M, Galkina EV. Functional role of B cells in atherosclerosis. Cells. 2021;10: 270. doi: 10.3390/cells10020270 33572939 PMC7911276

[7] Danesh J, Wheeler JG, Hirschfield GM, Eda S, Eiriksdottir G, Rumley A, et al. C-reactive protein and other circulating markers of inflammation in the prediction of coronary heart disease. N Engl J Med. 2004;350: 1387–1397. doi: 10.1056/NEJMoa032804 15070788

[8] Rost NS, Wolf PA, Kase CS, Kelly-Hayes M, Silbershatz H, Massaro JM, et al. Plasma concentration of C-reactive protein and risk of ischemic stroke and transient ischemic attack: the Framingham Study. Stroke. 2001;32: 2575–2579. doi: 10.1161/hs1101.098151 11692019

[9] Sabatine MS, Morrow DA, Cannon CP, Murphy SA, Demopoulos LA, DiBattiste PM, et al. Relationship between baseline white blood cell count and degree of coronary artery disease and mortality in patients with acute coronary syndromes: a TACTICS-TIMI 18 (Treat Angina with Aggrastat and determine Cost of Therapy with an Invasive or Conservative Strategy- Thrombolysis in myocardial infarction 18 trial) substudy. J Am Coll Cardiol. 2002;40: 1761–1768. doi: 10.1016/s0735-1097(02)02484-1 12446059

[10] Shen H, Dai Z, Wang M, Gu S, Xu W, Xu G, et al. Preprocedural neutrophil to albumin ratio predicts in-stent restenosis following carotid angioplasty and stenting. J Stroke Cerebrovasc Dis. 2019;28: 2442–2447. doi: 10.1016/j.jstrokecerebrovasdis.2019.06.027 31303439

[11] Hu B, Yang XR, Xu Y, Sun YF, Sun C, Guo W, et al. Systemic immune-inflammation index predicts prognosis of patients after curative resection for hepatocellular carcinoma. Clin Cancer Res. 2014;20: 6212–6222. doi: 10.1158/1078-0432.CCR-14-0442 25271081

[12] Yang YL, Wu CH, Hsu PF, Chen SC, Huang SS, Chan WL, et al. Systemic immune-inflammation index (SII) predicted clinical outcome in patients with coronary artery disease. Eur J Clin Invest. 2020;50: e13230. doi: 10.1111/eci.13230 32291748

[13] Li LH, Chen CT, Chang YC, Chen YJ, Lee IH, How CK. Prognostic role of neutrophil-to-lymphocyte ratio, platelet-to-lymphocyte ratio, and systemic immune inflammation index in acute ischemic stroke: a STROBE-compliant retrospective study. Medicine (Baltimore). 2021;100: e26354. doi: 10.1097/MD.0000000000026354 34160404 PMC8238279

[14] Keskin M, Öcal L, Cerşit S, Yılmaz C, Küp A, Çelik M, et al. The Predictive role of a novel risk index in patients undergoing carotid artery stenting: systemic immune-inflammation Index. J Stroke Cerebrovasc Dis. 2021;30: 105955. doi: 10.1016/j.jstrokecerebrovasdis.2021.105955 34242858

[15] Gurm HS, Yadav JS, Fayad P, Katzen BT, Mishkel GJ, Bajwa TK, et al. Long-term results of carotid stenting versus endarterectomy in high-risk patients. N Engl J Med. 2008;358: 1572–1579. doi: 10.1056/NEJMoa0708028 18403765

[16] Aboyans V, Ricco JB, Bartelink MEL, Björck M, Brodmann M, Cohnert T, et al. 2017 ESC Guidelines on the Diagnosis and Treatment of Peripheral Arterial Diseases, in collaboration with the European Society for Vascular Surgery (ESVS): document covering atherosclerotic disease of extracranial carotid and vertebral, mesenteric, renal, upper and lower extremity arteries Endorsed by: the European Stroke Organization (ESO) The Task Force for the Diagnosis and Treatment of Peripheral Arterial Diseases of the European Society of Cardiology (ESC) and of the European Society for Vascular Surgery (ESVS). Eur Heart J. 2018;39: 763–816. doi: 10.1093/eurheartj/ehx095 28886620

[17] Brott TG, Halperin JL, Abbara S, Bacharach JM, Barr JD, Bush RL, et al. 2011 ASA/ACCF/AHA/AANN/AANS/ACR/ASNR/CNS/SAIP/SCAI/SIR/SNIS/SVM/SVS guideline on the management of patients with extracranial carotid and vertebral artery disease: executive summary. Stroke. 2011;42: e420–e463. doi: 10.1161/STR.0b013e3182112d08 21282494

[18] Saw J, Gurm HS, Fathi RB, Bhatt DL, Abou-Chebl A, Bajzer C, et al. Effect of chronic kidney disease on outcomes after carotid artery stenting. Am J Cardiol. 2004;94: 1093–1096. doi: 10.1016/j.amjcard.2004.06.078 15476637

[19] Liberale L, Badimon L, Montecucco F, Lüscher TF, Libby P, Camici GG. Inflammation, aging, and cardiovascular disease: JACC review topic of the week. J Am Coll Cardiol. 2022;79: 837–847. doi: 10.1016/j.jacc.2021.12.017 35210039 PMC8881676

[20] Cybulsky MI, Gimbrone MA Jr. Endothelial expression of a mononuclear leukocyte adhesion molecule during atherogenesis. Science. 1991;251: 788–791. doi: 10.1126/science.1990440 1990440

[21] Li Y, Jia H, Yu W, Xu Y, Li X, Li Q, et al. Nomograms for predicting prognostic value of inflammatory biomarkers in colorectal cancer patients after radical resection. Int J Cancer. 2016;139: 220–231. doi: 10.1002/ijc.30071 26933932

[22] Silvestre-Roig C, Braster Q, Ortega-Gomez A, Soehnlein O. Neutrophils as regulators of cardiovascular inflammation. Nat Rev Cardiol. 2020;17: 327–340. doi: 10.1038/s41569-019-0326-7 31996800

[23] Buffon A, Biasucci LM, Liuzzo G, D’Onofrio G, Crea F, Maseri A. Widespread coronary inflammation in unstable angina. N Engl J Med. 2002;347: 5–12. doi: 10.1056/NEJMoa012295 12097534

[24] Wheeler JG, Mussolino ME, Gillum RF, Danesh J. Associations between differential leucocyte count and incident coronary heart disease: 1764 incident cases from seven prospective studies of 30,374 individuals. Eur Heart J. 2004;25: 1287–1292. doi: 10.1016/j.ehj.2004.05.002 15288155

[25] Grau AJ, Boddy AW, Dukovic DA, Buggle F, Lichy C, Brandt T, et al. Leukocyte count as an independent predictor of recurrent ischemic events. Stroke. 2004;35: 1147–1152. doi: 10.1161/01.STR.0000124122.71702.64 15017013

[26] Hotchkiss RS, Karl IE. The pathophysiology and treatment of sepsis. N Engl J Med. 2003;348: 138–150. doi: 10.1056/NEJMra021333 12519925

[27] Pellizzon GG, Dixon SR, Stone GW, Cox DA, Mattos L, Boura JA, et al. Relation of admission white blood cell count to long-term outcomes after primary coronary angioplasty for acute myocardial infarction (The Stent PAMI Trial). Am J Cardiol. 2003;91: 729–731. doi: 10.1016/s0002-9149(02)03416-1 12633810

[28] Mueller C, Neumann FJ, Hochholzer W, Trenk D, Zeller T, Perruchoud AP, et al. The impact of platelet count on mortality in unstable angina/non-ST-segment elevation myocardial infarction. Am Heart J. 2006;151: 1214.e1–1214.e7. doi: 10.1016/j.ahj.2006.03.011 16781221

[29] Gröschel K, Ernemann U, Larsen J, Knauth M, Schmidt F, Artschwager J, et al. Preprocedural C-reactive protein levels predict stroke and death in patients undergoing carotid stenting. AJNR Am J Neuroradiol. 2007;28: 1743–1746. doi: 10.3174/ajnr.A0650 17885237 PMC8134178

[30] Stone PA, Thompson SN, Khan M, Northfield E, Schillinger R, Skaff P. The impact of biochemical markers on major adverse cardiovascular events and contralateral carotid artery stenosis progression following carotid interventions. Ann Vasc Surg. 2017;38: 144–150. doi: 10.1016/j.avsg.2016.08.004 27546852

[31] Li Y, Zhong X, Cheng G, Zhao C, Zhang L, Hong Y, et al. Hs-CRP and all-cause, cardiovascular, and cancer mortality risk: a meta-analysis. Atherosclerosis. 2017;259: 75–82. doi: 10.1016/j.atherosclerosis.2017.02.003 28327451

[32] Aronow HD, Shishehbor M, Davis DA, Katzan IL, Bhatt DL, Bajzer CT, et al. Leukocyte count predicts microembolic Doppler signals during carotid stenting: a link between inflammation and embolization. Stroke. 2005;36: 1910–1914. doi: 10.1161/01.STR.0000177610.33478.65 16100016

